# Pituitary Adenylate Cyclase-Activating Polypeptide in Learning and Memory

**DOI:** 10.3389/fncel.2021.663418

**Published:** 2021-06-22

**Authors:** Marieke R. Gilmartin, Nicole C. Ferrara

**Affiliations:** ^1^Marquette University, Milwaukee, WI, United States; ^2^Rosalind Franklin University of Medicine and Science, North Chicago, IL, United States

**Keywords:** PACAP, learning, working memory, fear, anxiety, cognition, sex, PTSD

## Abstract

Pituitary adenylate cyclase-activating polypeptide (PACAP) is a highly conserved neuropeptide that regulates neuronal physiology and transcription through Gs/Gq-coupled receptors. Its actions within hypothalamic, limbic, and mnemonic systems underlie its roles in stress regulation, affective processing, neuroprotection, and cognition. Recently, elevated PACAP levels and genetic disruption of PAC1 receptor signaling in humans has been linked to maladaptive threat learning and pathological stress and fear in post-traumatic stress disorder (PTSD). PACAP is positioned to integrate stress and memory in PTSD for which memory of the traumatic experience is central to the disorder. However, PACAP’s role in memory has received comparatively less attention than its role in stress. In this review, we consider the evidence for PACAP-PAC1 receptor signaling in learning and plasticity, discuss emerging data on sex differences in PACAP signaling, and raise key questions for further study toward elucidating the contribution of PACAP to adaptive and maladaptive fear learning.

## Introduction

The salient experiences of our daily life create memories that form the narrative of our self. These memories enrich our connections with others and guide our decisions and behavior, but if born out of pain and trauma, can be debilitating. Intrusive, terrifying memories that are difficult to extinguish or that generalize to non-threatening situations are the hallmark of post-traumatic stress disorder (PTSD), a complex disorder with a lifetime prevalence of about 8% (Kessler et al., 1995; Kilpatrick et al., 2013). PTSD is characterized by altered stress reactivity, generalized fear to non-threatening cues and situations, and is more prevalent in females (Kessler et al., 1995; Kilpatrick et al., 2013). Recently, the neuropeptide pituitary adenylate cyclase-activating polypeptide (PACAP) has been linked to PTSD (Ressler et al., 2011; Almli et al., 2013; Uddin et al., 2013). This link has been attributed to the role that PACAP plays in regulating the somatic and affective components of chronic stress, discussed in excellent reviews (Hammack and May, 2015; King et al., 2017b; Miles and Maren, 2019). However, PACAP’s role in memory has received comparatively less attention yet may also contribute to PTSD for which memory of the traumatic experience is central to the disorder. Here, we briefly review the literature supporting a role for PACAP in memory formation and discuss avenues for further investigation.

## PACAP, an Overview

Pituitary adenylate cyclase-activating polypeptide is a highly conserved pleiotropic neuropeptide in the vasoactive intestinal peptide (VIP)/secretin/glucagon family that modulates several physiological functions in the periphery and central nervous system *via* class B G-protein coupled receptors (Miyata et al., 1989, 1990; Arimura et al., 1991; Piggins et al., 1996; Arimura, 1998; Vaudry et al., 2009). In the brain, the 38-amino acid form of PACAP predominates (Arimura, 1998), and PACAP-38 and its receptors are widely expressed in circuits involved in memory, stress, and affect (Shioda et al., 1997; Hannibal, 2002; Joo et al., 2004; Condro et al., 2016). PACAP modulates neuronal function *via* the PACAP-specific high-affinity receptor PAC1 and the VPAC1 and VPAC2 receptors, which have similar affinity for PACAP and VIP. These receptors are coupled to Gαs, but PAC1 can also signal through Gαq (Spengler et al., 1993; Dickson and Finlayson, 2009; Vaudry et al., 2009; Harmar et al., 2012). Thus, PACAP can regulate the neuronal excitability and synaptic plasticity underlying memory and cognition through a diverse set of cAMP-mediated intracellular signaling (for a review see Johnson et al., 2019).

## Distributed Memory Systems

A role for PACAP in learning and memory was evident from early examinations of mice lacking the PAC1 receptor (PAC1R). Genetic deletion of PAC1R either globally or in the forebrain produced mild to severe impairments in certain forms of hippocampus-dependent learning (Sauvage et al., 2000; Otto et al., 2001a, b). Contextual fear conditioning, an associative paradigm in which subjects learn to associate the spatial configuration of environmental cues with a footshock, was impaired, while other hippocampus-dependent learning tasks, the Morris water maze and social transmission of food preference were unaffected. Hippocampus-independent cued fear conditioning was also unaffected by the loss of PAC1R signaling in the same study. Global knockouts displayed anxiety-like behavior and altered stress reactivity, which is consistent with PACAP’s role in HPA axis regulation, but which could affect the conditional response of freezing used to assess memory in rodent fear conditioning. Thus, it is important that the forebrain-specific knockouts, which showed the same memory deficit, did not differ from wild-type controls in locomotor activity or anxiety-like activity in the open field or elevated plus maze (Otto et al., 2001b). Mice lacking the PACAP peptide also showed impaired contextual fear memory as well as deficits in novel object recognition (Takuma et al., 2014). However, these mice exhibit a wide range of altered behavior (reviewed in Hashimoto et al., 2006). Some of these behaviors could be ameliorated with environmental enrichment early in life, but not in adulthood, suggesting that PACAP’s role in neural development may contribute to abnormal behaviors in PACAP deficient mice (Ishihama et al., 2010; Takuma et al., 2014). Nonetheless, exogenous delivery of PACAP intracerebroventricularly into adult rats enhanced the consolidation of a passive avoidance memory at low doses (Sacchetti et al., 2001) and temporarily impaired contextual fear memory at high doses (Meloni et al., 2016, 2018). These studies demonstrate a role for forebrain PACAP in contextual fear learning and suggest that PAC1R signaling may be preferentially engaged by aversive events.

More recently, behavioral pharmacology studies have identified specific brain regions where PACAP contributes to aversive memory or its extinction. These include the hippocampus, amygdala, and prelimbic cortex. In the hippocampus, the consolidation of contextual fear memory was enhanced by PACAP and impaired by the PAC1R antagonist PACAP6-38, when injected immediately after training (Schmidt et al., 2015). Hippocampal PACAP also contributes to fear extinction, while amygdala PACAP is needed for contextual fear memory but not its extinction (Schmidt et al., 2015). The subregional specificity of PACAP’s effects in the hippocampus remain to be determined. The hippocampus is functionally heterogeneous with unique output connectivity along its dorsal-ventral axis (Dong et al., 2009). These studies targeted dorsal hippocampus and given the importance of ventral hippocampus to affective behavior and learning, future work should examine ventral hippocampal PACAP. PACAP also participates in the formation of trace fear memory, a form of cued fear learning dependent on the prelimbic cortex and hippocampus in addition to the amygdala (Kirry et al., 2018). Trace conditioning requires the association of a cue and shock separated in time, and linking these events requires sustained neuronal activity in prelimbic cortex (Baeg et al., 2001; Gilmartin and McEchron, 2005; Gilmartin et al., 2013b, 2014). Prelimbic injection of PACAP6-38 prior to training impaired the formation of the cued memory in females, but not males (Kirry et al., 2018). This sex difference, discussed below, may provide insight into the genetic link between PAC1R and PTSD, for which women with a genetic polymorphism in the PAC1R gene exhibit enhanced reactivity to threat-predictive cues (Ressler et al., 2011). The prefrontal cortex is also needed for aspects of contextual fear learning (Gilmartin and Helmstetter, 2010; Gilmartin et al., 2013a; Rozeske et al., 2015; Heroux et al., 2017; Twining et al., 2020), but PAC1R antagonism did not affect the contextual fear memory formed alongside the cued fear memory in trace conditioning (Kirry et al., 2018). Nor did the manipulation affect a non-aversive spatial-working memory T-maze task in either sex. These data suggest that PAC1R signaling contributes to prefrontal mechanisms of working memory or sustained attention required for predicting threat based on available cues. Together, these studies demonstrate a role for PACAP signaling in learning and memory and point to site-specific engagement of PACAP in cued and contextual fear.

## Cellular and Synaptic Physiology

The regulation of synaptic glutamatergic signaling and the production of new proteins for long-term synaptic stabilization are the basis of the cellular consolidation of memory, a process that generally concludes within a few hours after training (McGaugh, 2000; Asok et al., 2019). Adenylate cyclase/cAMP-driven intracellular signaling leading to CREB-mediated gene transcription is critical for the formation of long-term memories (Kandel et al., 2014; Asok et al., 2019). PACAP’s namesake ability to activate these signaling cascades underlies its role in memory. In hippocampal circuits, PACAP modulates synaptic and evoked NMDA- and AMPA-mediated currents *via* PKA or PLC/PKC (Roberto and Brunelli, 2000; Roberto et al., 2001; Ciranna and Cavallaro, 2003; Yaka et al., 2003; Macdonald et al., 2005; Costa et al., 2009; Pecoraro et al., 2017). PACAP-dependent phosphorylation of NMDA receptor subunits promotes mossy fiber long-term potentiation (LTP), a cellular correlate of learning and memory, and LTP is impaired in mice lacking PACAP or PAC1R (Otto et al., 2001a; Matsuyama et al., 2003). PACAP’s effects on plasticity are dose-dependent, with higher doses exerting inhibitory effects on hippocampal synaptic transmission *via* VPAC signaling (Costa et al., 2009), which reflects its dose-dependent effects on hippocampus-dependent fear memory (Sacchetti et al., 2001; Meloni et al., 2016). It is important to note that temperature is an important factor in the physiological investigation of PACAP. While lower temperatures (e.g., 21–24°C) are useful for slowing down the fast ion channel kinetics linked to PAC1R activation (Johnson et al., 2019), they can alter other measures of excitability and interfere with PAC1R endosomal signaling (Merriam et al., 2013). Moreover, the majority of existing hippocampal work has focused on CA1 and CA3, but PAC1R is expressed also in the dentate gyrus (Jaworski and Proctor, 2000; Joo et al., 2004). Recent work has shown that PACAP-PAC1R activation drives CREB-mediated transcription and promotes excitability of DG granule cells (Johnson et al., 2019; Johnson R. et al., 2020; Johnson R. L. et al., 2020).

Pituitary adenylate cyclase-activating polypeptide has dose-, receptor-, and circuit-specific effects on physiology in other brain areas, which highlights the potential ways in which PACAP can affect learning within a distributed network. In the amygdala, PACAP increases AMPA-mediated currents at BLA-CeA synapses *via* VPAC1 (Cho et al., 2012) and increases GABA release *via* PAC1R (Varodayan et al., 2019), mechanisms that underlie affective behavioral responses to chronic stress or pain. In the central nucleus of the amygdala, fear conditioning drives expression of the plasticity-related protein Arc and intracerebroventricular infusion of PACAP enhances this expression (Meloni et al., 2018). Arc, whose translation is regulated by PKA activity (Bloomer et al., 2008), is critical to several cellular processes supporting memory and cognition (e.g., Nikolaienko et al., 2018). PACAP’s contribution to synaptic plasticity in cortical systems is not clear. Given the selective role for prelimbic PAC1R signaling in trace cued, but not contextual, memory (Kirry et al., 2018), PACAP may act on working-memory or sustained attention mechanisms. One candidate mechanism is the regulation of GluN2B-containing NMDARs, which promote recurrent activity in cortical circuits (Wang et al., 2013) and which are needed for trace cued, but not contextual fear learning in the prefrontal cortex and hippocampus (Gao et al., 2010; Gilmartin et al., 2013a). PACAP has been shown to phosphorylate the GluN2B subunit in the hippocampus and hypothalamus to regulate glutamatergic signaling (Yaka et al., 2003; Resch et al., 2014).

Pituitary adenylate cyclase-activating polypeptide can also influence memory *via* modulation of intrinsic physiology (see Open Questions) and *via* developmental maturation of memory circuits (reviewed in Shen et al., 2013). For example, developmental knockout of VPAC2 which is sensitive to both VIP and PACAP prevented the formation of fear extinction memory in adulthood (Ago et al., 2017). Importantly, VPAC2-KO mice had reduced cell size and dendritic branching in the prelimbic cortex, morphological changes similar to those observed after chronic corticosterone exposure and chronic stress, conditions which also produce fear extinction deficits (Wellman, 2001; Radley et al., 2004, 2006; Moench and Wellman, 2017; reviewed in Wellman et al., 2020). Whether impaired extinction in VPAC2-KO mice is a consequence of altered prefrontal morphology or lack of PACAP/VIP signaling at VPAC2 in the prefrontal-amygdala circuit during extinction learning remains to be determined. Nonetheless, these neuropeptides are necessary for proper neural maturation, and pathological disruptions in PACAP signaling during critical developmental windows could thus affect adult cognition by altering the development of memory-related circuits. Although we are only beginning to elucidate the diverse mechanisms by which PACAP affects learning and memory, the significant work on the diversity of PACAP signaling in stress-related behaviors provides a useful foundation for rapid progress in this effort (Hammack and May, 2015; Johnson et al., 2019; Ferrara and Gilmartin, 2020).

## Sex Differences

The work above implicates PACAP and PAC1R in neuroplasticity and memory and suggests that endogenous PACAP released during salient, aversive events contributes to memories for threat-predictive cues and contexts. Therefore, vulnerabilities in PACAP signaling may contribute to pathological fear in PTSD through its roles in learning and in mediating traumatic stress responses. Further investigation of the PACAP-PTSD link requires inclusion of female subjects, not only because females have largely been excluded from preclinical study, but also because of a sex-specific link between PACAP and PTSD. A single-nucleotide polymorphism (SNP) in the *adcyap1r1* gene encoding PAC1R is associated with symptom severity in women, but not men; altered DNA methylation of the same gene is associated with PTSD in both sexes (Ressler et al., 2002; Almli et al., 2013). The risk allele for PTSD is also associated with symptom severity in GAD females, but not males (Ross et al., 2020). The SNP is located in an estrogen-response element (ERE), and the nucleotide change interferes with estradiol-estrogen receptor alpha binding the ERE, leading to a decrease in receptor expression (Mercer et al., 2016). This points to a potential mechanism of vulnerability in women. Indeed, several labs are actively examining how PACAP in the BNST and hypothalamic stress circuits regulates the affective response to stress in male and female rodents (King et al., 2017a, b; Ramikie and Ressler, 2018), and additional mechanistic insight may come from examination of sex differences in autonomic regulation by PACAP (Nakamachi et al., 2016). This work complements that of Bangasser, Valentino, and others detailing sex differences in the corticotropin-releasing factor (CRF) system and brainstem arousal systems [for excellent reviews, see Bangasser and Valentino (2014); Valentino and Bangasser (2016); Bangasser et al. (2019)].

Little is known about sex differences in PACAP’s contribution to memory. Recent work showed that prelimbic PACAP participates in trace cued fear learning in females, but not males, and that mRNA levels for PAC1R are higher in females than males and further modulated by the estrous cycle (Kirry et al., 2018). Recently, Rajbhandari et al. (2021) reported increased fear generalization and impaired extinction in males, but not females, following viral deletion of PAC1R in the medial intercalated cells of the amygdala, a region involved in the suppression of fear following extinction (Duvarci and Pare, 2014). Females in that study showed a reduced asymptotic level of fear during acquisition. These behavioral results suggest that PACAP signaling exerts sex and region-specific modulation of fear memories. In humans, the PTSD-related PAC1R risk allele is associated with enhanced startle to threat-related cues, impaired fear and safety discrimination, and altered hippocampal and amygdala reactivity in fear conditioning (Ressler et al., 2011; Jovanovic et al., 2013; Stevens et al., 2014). In children, females with the risk allele showed enhanced fear responding to threat-related cues 1 year after conditioning (Jovanovic et al., 2020). While these clinical studies implicate associative learning processes in the PAC1R-PTSD link, it is difficult to distinguish the unique contributions of associative learning vs stress reactivity. Preclinical investigations are critical in this endeavor.

## Open Questions

Pituitary adenylate cyclase-activating polypeptide contributes to learning and memory under salient, usually aversive conditions. The diversity of its neural function places this pleiotropic signaling peptide in the company of several peptide factors, such as CRF and estradiol, that have a wide range of function beyond that for which they were initially characterized (Hupalo et al., 2019; Taxier et al., 2020). This diversity likely underlies the influence that PACAP dysregulation appears to have in psychiatric illness, but also makes it challenging to determine the nature of that relationship. Here, we raise a few open questions to guide further investigation.

### Open Question 1: Is PACAP’s Role in Learning Selective for Aversive Episodic Memory?

Disruption of PAC1R signaling affects the formation of associative fear memories dependent on episodic memory systems, such as contextual fear or trace fear conditioning, but leaves standard delay cued conditioning and spatial learning largely intact. Interestingly, the *amnesiac* gene in drosophila, which codes for a putative PACAP homolog, AMN, is necessary for odor-shock associative memory (Quinn et al., 1979; Feany and Quinn, 1995; Turrel et al., 2018). While the receptor target(s) by which AMN mediates memory is unclear, the parallels in these behavioral observations raise the possibility that PACAP is selective for aversive learning; however, its role in appetitive or other non-aversive learning is largely untested. Thus, to determine PACAP selectivity to certain forms of memory, its contribution to non-aversive memory needs to be clarified. Importantly, PACAP and PAC1R manipulations in the mature circuit are needed to rule out learning deficits due to aberrant neural development of healthy memory circuits for which PACAP is implicated. To this point, the role for AMN in the development of associative memory systems was recently dissociated from its role in adult learning (Turrel et al., 2018).

### Open Question 2: What Aspect of an Aversive Experience Recruits PACAP Signaling?

Pituitary adenylate cyclase-activating polypeptide is mobilized by repeated or chronic stressors or by persistent neuropathic pain states (Dickinson and Fleetwood-Walker, 1999; Mustafa, 2013), but release conditions in learning networks during relatively brief aversive events in fear conditioning are unknown. PACAP-expressing cells are found throughout learning circuits (Hannibal, 2002; Condro et al., 2016), many of which robustly respond to shock delivery. One possibility is that the aversive shock reinforcer may trigger the co-release of PACAP at glutamatergic terminals, facilitating cAMP-mediated signaling in support of robust fear memory. Alternatively, any sufficiently salient or arousing experience may mobilize PACAP and promote the consolidation of memory. The development of tools that allow accurate measurement of peptide release *in vivo* provide the circuit-level resolution needed to determine when and where PACAP is released during learning (Muller et al., 2014; Al-Hasani et al., 2018).

### Open Question 3: What Are the Unique Peptide-Receptor Signaling Contributions to Learning and Memory?

The unique contributions of PACAP and VIP at PAC1 and VPAC1/2 receptors in learning and memory are poorly understood. For instance, VPAC2 receptors have partially overlapping distribution patterns in several regions important for learning and memory (Lee et al., 2010), and developmental manipulation of VPAC2 affects extinction learning in adulthood (Ago et al., 2017). Pharmacological dissociation of PACAP actions at PAC1R and VPAC2 is difficult as both are a target of the PAC1R antagonist PACAP6-38. Plus, as mentioned earlier, mechanistic dissection of these peptides *in vitro* is sensitive to experimental parameters such as temperature. Thus, new small molecule antagonists as well as gene-editing tools will be invaluable in revealing the rich complexity of mnemonic regulation by these peptides.

### Open Question 4: Does PACAP Bias Allocation of Specific Circuits Into Memory?

One intriguing, but speculative, role for PACAP in memory is to bias the allocation of cells or circuits into memory as a consequence of psychological stress or aversive experience (Kondo et al., 1997). Neuronal excitability at the time of learning determines which neurons are allocated to the memory trace (Yiu et al., 2014; Cai et al., 2016; Sehgal et al., 2018; Josselyn and Tonegawa, 2020). In the amygdala, cells with reduced afterhyperpolarization (AHP) and increased CREB phosphorylation immediately prior to training are recruited into a fear memory (Yiu et al., 2014). PACAP reduces the slow AHP calcium-activated potassium current sI_*AHP*_ *via* cAMP/PKA to increase excitability and action potential firing in hippocampal CA1 and neocortical neurons (Hu et al., 2011; Taylor et al., 2014). PACAP-elicited firing and PKA-dependent phosphorylation of CREB drive CREB-mediated transcription (Baxter et al., 2011). PACAP affects excitability and firing in a circuit-specific manner (e.g., Hurley et al., 2019) and intrinsic properties can be modified by experience (Sehgal et al., 2013; Dunn and Kaczorowski, 2019). Thus, PACAP released during stressful events or elevated PACAP following chronic stress or pain could influence which circuits are recruited into a memory trace. Addressing this possibility will shed light on how altered PACAP-PAC1R signaling in susceptible individuals corresponds with altered threat-associated memory in PTSD.

## Concluding Remarks

Pituitary adenylate cyclase-activating polypeptide is a pleiotropic neuropeptide whose diverse signaling underlies its diversity of function including neural development, neuroprotection, stress regulation, autonomic activation, affective behavior, and memory. This in turn highlights the therapeutic potential of this peptide and receptor modulations, a potential that is discussed in this Research Topic and reflected in recent efforts to develop non-peptide small molecule compounds to selectively target PACAP receptors (Beebe et al., 2008; Takasaki et al., 2018). The link between PACAP-PAC1R and pathological fear learning and stress dysregulation in PTSD suggests another potential therapeutic use for such treatments. Realization of this potential requires continued efforts to address the role of PACAP in learning and in the complex interactions of stress and sex on memory.

## Author Contributions

MG and NF wrote and edited the manuscript. Both authors contributed to the article and approved the submitted version.

## Conflict of Interest

The authors declare that the research was conducted in the absence of any commercial or financial relationships that could be construed as a potential conflict of interest.

## References

[B1] Ago Y. Hayata-Takano A. Kawanai T. Yamauchi R. Takeuchi S. Cushman J. D. (2017). Impaired extinction of cued fear memory and abnormal dendritic morphology in the prelimbic and infralimbic cortices in VPAC2 receptor (VIPR2)-deficient mice. *Neurobiol. Learn. Mem.* 145 222–231. 10.1016/j.nlm.2017.10.010 29030297 PMC5698124

[B2] Al-Hasani R. Wong J. T. Mabrouk O. S. McCall J. G. Schmitz G. P. Porter-Stransky K. A. (2018). In vivo detection of optically-evoked opioid peptide release. *eLife* 7:e36520. 30175957 10.7554/eLife.36520 PMC6135606

[B3] Almli L. M. Mercer K. B. Kerley K. Feng H. Bradley B. Conneely K. N. (2013). ADCYAP1R1 genotype associates with post-traumatic stress symptoms in highly traumatized African-American females. *Am. J. Med. Genet. B Neuropsychiatr. Genet.* 162B 262–272. 10.1002/ajmg.b.32145 23505260 PMC3738001

[B4] Arimura A. (1998). Perspectives on pituitary adenylate cyclase activating polypeptide (PACAP) in the neuroendocrine, endocrine, and nervous systems. *Jpn. J. Physiol.* 48 301–331. 10.2170/jjphysiol.48.301 9852340

[B5] Arimura A. Somogyvari-Vigh A. Miyata A. Mizuno K. Coy D. H. Kitada C. (1991). Tissue distribution of PACAP as determined by RIA: highly abundant in the rat brain and testes. *Endocrinology* 129 2787–2789. 10.1210/endo-129-5-2787 1935809

[B6] Asok A. Leroy F. Rayman J. B. Kandel E. R. (2019). Molecular mechanisms of the memory trace. *Trends Neurosci.* 42 14–22. 10.1016/j.tins.2018.10.005 30391015 PMC6312491

[B7] Baeg E. H. Kim Y. B. Jang J. Kim H. T. Mook-Jung I. Jung M. W. (2001). Fast spiking and regular spiking neural correlates of fear conditioning in the medial prefrontal cortex of the rat. *Cereb. Cortex* 11 441–451. 10.1093/cercor/11.5.441 11313296

[B8] Bangasser D. A. Valentino R. J. (2014). Sex differences in stress-related psychiatric disorders: neurobiological perspectives. *Front. Neuroendocrinol.* 35 303–319. 10.1016/j.yfrne.2014.03.008 24726661 PMC4087049

[B9] Bangasser D. A. Eck S. R. Ordones Sanchez E. (2019). Sex differences in stress reactivity in arousal and attention systems. *Neuropsychopharmacology* 44 129–139. 10.1038/s41386-018-0137-2 30022063 PMC6235989

[B10] Baxter P. S. Martel M. A. McMahon A. Kind P. C. Hardingham G. E. (2011). Pituitary adenylate cyclase-activating peptide induces long-lasting neuroprotection through the induction of activity-dependent signaling via the cyclic AMP response element-binding protein-regulated transcription co-activator 1. *J. Neurochem.* 118 365–378. 10.1111/j.1471-4159.2011.07330.x 21623792 PMC3557719

[B11] Beebe X. Darczak D. Davis-Taber R. A. Uchic M. E. Scott V. E. Jarvis M. F. (2008). Discovery and SAR of hydrazide antagonists of the pituitary adenylate cyclase-activating polypeptide (PACAP) receptor type 1 (PAC1-R). *Bioorg. Med. Chem. Lett.* 18 2162–2166. 10.1016/j.bmcl.2008.01.052 18272364

[B12] Bloomer W. A. VanDongen H. M. VanDongen A. M. (2008). Arc/Arg3.1 translation is controlled by convergent N-methyl-D-aspartate and Gs-coupled receptor signaling pathways. *J. Biol. Chem.* 283 582–592. 10.1074/jbc.m702451200 17981809

[B13] Cai D. J. Aharoni D. Shuman T. Shobe J. Biane J. Song W. (2016). A shared neural ensemble links distinct contextual memories encoded close in time. *Nature* 534 115–118. 10.1038/nature17955 27251287 PMC5063500

[B14] Cho J. H. Zushida K. Shumyatsky G. P. Carlezon W. A. Meloni E. G. Bolshakov V. Y. (2012). Pituitary adenylate cyclase-activating polypeptide induces postsynaptically expressed potentiation in the intra-amygdala circuit. *J. Neurosci.* 32 14165–14177. 10.1523/jneurosci.1402-12.2012 23055486 PMC3490216

[B15] Ciranna L. Cavallaro S. (2003). Opposing effects by pituitary adenylate cyclase-activating polypeptide and vasoactive intestinal peptide on hippocampal synaptic transmission. *Exp. Neurol.* 184 778–784. 10.1016/s0014-4886(03)00300-5 14769370

[B16] Condro M. C. Matynia A. Foster N. N. Ago Y. Rajbhandari A. K. Van C. (2016). High-resolution characterization of a PACAP-EGFP transgenic mouse model for mapping PACAP-expressing neurons. *J. Comp. Neurol.* 524 3827–3848. 10.1002/cne.24035 27197019 PMC5063673

[B17] Costa L. Santangelo F. Li Volsi G. Ciranna L. (2009). Modulation of AMPA receptor-mediated ion current by pituitary adenylate cyclase-activating polypeptide (PACAP) in CA1 pyramidal neurons from rat hippocampus. *Hippocampus* 19 99–109. 10.1002/hipo.20488 18727050

[B18] Dickinson T. Fleetwood-Walker S. M. (1999). VIP and PACAP: very important in pain? *Trends Pharmacol. Sci.* 20 324–329. 10.1016/s0165-6147(99)01340-1 10431211

[B19] Dickson L. Finlayson K. (2009). VPAC and PAC receptors: from ligands to function. *Pharmacol. Ther.* 121 294–316. 10.1016/j.pharmthera.2008.11.006 19109992

[B20] Dong H. W. Swanson L. W. Chen L. Fanselow M. S. Toga A. W. (2009). Genomic-anatomic evidence for distinct functional domains in hippocampal field CA1. *Proc. Natl. Acad. Sci. U. S. A.* 106 11794–11799. 10.1073/pnas.0812608106 19561297 PMC2710698

[B21] Dunn A. R. Kaczorowski C. C. (2019). Regulation of intrinsic excitability: roles for learning and memory, aging and Alzheimer’s disease, and genetic diversity. *Neurobiol. Learn. Mem.* 164:107069. 10.1016/j.nlm.2019.107069 31442579 PMC6752224

[B22] Duvarci S. Pare D. (2014). Amygdala microcircuits controlling learned fear. *Neuron* 82 966–980. 10.1016/j.neuron.2014.04.042 24908482 PMC4103014

[B23] Feany M. B. Quinn W. G. (1995). A neuropeptide gene defined by the Drosophila memory mutant amnesiac. *Science* 268 869–873. 10.1126/science.7754370 7754370

[B24] Ferrara N. C. Gilmartin M. R. (2020). “Pituitary adenylate cyclase-activating polypeptide (PACAP) in stress, pain, and learning,” in *Handbook of Amygdala Structure and Function*, ed. Urban J. H. (Amsterdam: Elsevier).

[B25] Gao C. Gill M. B. Tronson N. C. Guedea A. L. Guzman Y. F. Huh K. H. (2010). Hippocampal NMDA receptor subunits differentially regulate fear memory formation and neuronal signal propagation. *Hippocampus* 20 1072–1082. 10.1002/hipo.20705 19806658 PMC2891656

[B26] Gilmartin M. R. Helmstetter F. J. (2010). Trace and contextual fear conditioning require neural activity and NMDA receptor-dependent transmission in the medial prefrontal cortex. *Learn. Mem.* 17 289–296. 10.1101/lm.1597410 20504949 PMC2884289

[B27] Gilmartin M. R. McEchron M. D. (2005). Single neurons in the medial prefrontal cortex of the rat exhibit tonic and phasic coding during trace fear conditioning. *Behav. Neurosci.* 119 1496–1510. 10.1037/0735-7044.119.6.1496 16420154

[B28] Gilmartin M. R. Balderston N. L. Helmstetter F. J. (2014). Prefrontal cortical regulation of fear learning. *Trends Neurosci.* 37 455–464. 10.1016/j.tins.2014.05.004 24929864 PMC4119830

[B29] Gilmartin M. R. Kwapis J. L. Helmstetter F. J. (2013a). NR2A-and NR2B-containing NMDA receptors in the prelimbic medial prefrontal cortex differentially mediate trace, delay, and contextual fear conditioning. *Learn. Mem.* 20 290–294. 10.1101/lm.030510.113 23676200 PMC3677083

[B30] Gilmartin M. R. Miyawaki H. Helmstetter F. J. Diba K. (2013b). Prefrontal activity links nonoverlapping events in memory. *J. Neurosci.* 33 10910–10914. 10.1523/jneurosci.0144-13.2013 23804110 PMC3693060

[B31] Hammack S. E. May V. (2015). Pituitary adenylate cyclase activating polypeptide in stress-related disorders: data convergence from animal and human studies. *Biol. Psychiatry* 78 167–177. 10.1016/j.biopsych.2014.12.003 25636177 PMC4461555

[B32] Hannibal J. (2002). Pituitary adenylate cyclase-activating peptide in the rat central nervous system: an immunohistochemical and in situ hybridization study. *J. Comp. Neurol.* 453 389–417. 10.1002/cne.10418 12389210

[B33] Harmar A. J. Fahrenkrug J. Gozes I. Laburthe M. May V. Pisegna J. R. (2012). Pharmacology and functions of receptors for vasoactive intestinal peptide and pituitary adenylate cyclase-activating polypeptide: IUPHAR review 1. *Br. J. Pharmacol.* 166 4–17. 10.1111/j.1476-5381.2012.01871.x 22289055 PMC3415633

[B34] Hashimoto H. Shintani N. Baba A. (2006). New insights into the central PACAPergic system from the phenotypes in PACAP- and PACAP receptor-knockout mice. *Ann. N. Y. Acad. Sci.* 1070 75–89. 10.1196/annals.1317.038 16888150

[B35] Heroux N. A. Robinson-Drummer P. A. Sanders H. R. Rosen J. B. Stanton M. E. (2017). Differential involvement of the medial prefrontal cortex across variants of contextual fear conditioning. *Learn. Mem.* 24 322–330. 10.1101/lm.045286.117 28716952 PMC5516685

[B36] Hu E. Demmou L. Cauli B. Gallopin T. Geoffroy H. Harris-Warrick R. M. (2011). VIP, CRF, and PACAP act at distinct receptors to elicit different cAMP/PKA dynamics in the neocortex. *Cereb. Cortex* 21 708–718. 10.1093/cercor/bhq143 20699230 PMC3041014

[B37] Hupalo S. Bryce C. A. Bangasser D. A. Berridge C. W. Valentino R. J. Floresco S. B. (2019). Corticotropin-releasing factor (CRF) circuit modulation of cognition and motivation. *Neurosci. Biobehav. Rev.* 103 50–59. 10.1016/j.neubiorev.2019.06.010 31212019 PMC6692202

[B38] Hurley M. M. Robble M. R. Callan G. Choi S. Wheeler R. A. (2019). Pituitary adenylate cyclase-activating polypeptide (PACAP) acts in the nucleus accumbens to reduce hedonic drive. *Int. J. Obes.* 43 928–932. 10.1038/s41366-018-0154-6 30082747 PMC6363914

[B39] Ishihama T. Ago Y. Shintani N. Hashimoto H. Baba A. Takuma K. (2010). Environmental factors during early developmental period influence psychobehavioral abnormalities in adult PACAP-deficient mice. *Behav. Brain Res.* 209 274–280. 10.1016/j.bbr.2010.02.009 20144662

[B40] Jaworski D. M. Proctor M. D. (2000). Developmental regulation of pituitary adenylate cyclase-activating polypeptide and PAC(1) receptor mRNA expression in the rat central nervous system. *Brain Res. Dev. Brain Res.* 120 27–39. 10.1016/s0165-3806(99)00192-3 10727727

[B41] Johnson G. C. May V. Parsons R. L. Hammack S. E. (2019). Parallel signaling pathways of pituitary adenylate cyclase activating polypeptide (PACAP) regulate several intrinsic ion channels. *Ann. N. Y. Acad. Sci.* 1455 105–112. 10.1111/nyas.14116 31162688 PMC6834876

[B42] Johnson R. L. Parsons G. C. May V. Hammack S. E. (2020). Pituitary adenylate cyclase-activating polypeptide-induced PAC1 receptor internalization and recruitment of MEK/ERK signaling enhance excitability of dentate gyrus granule cells. *Am. J. Physiol. Cell Physiol.* 318 C870–C878. 32186931 10.1152/ajpcell.00065.2020 PMC7294323

[B43] Johnson R. Parsons G. C. May V. Hammack S. E. (2020). The role of pituitary adenylate cyclase-activating polypeptide (PACAP) signaling in the hippocampal dentate Gyrus. *Front. Cell. Neurosci.* 14:111. 32425759 10.3389/fncel.2020.00111 PMC7203336

[B44] Joo K. M. Chung Y. H. Kim M. K. Nam R. H. Lee B. L. Lee K. H. (2004). Distribution of vasoactive intestinal peptide and pituitary adenylate cyclase-activating polypeptide receptors (VPAC1, VPAC2, and PAC1 receptor) in the rat brain. *J. Comp. Neurol.* 476 388–413. 10.1002/cne.20231 15282712

[B45] Josselyn S. A. Tonegawa S. (2020). Memory engrams: recalling the past and imagining the future. *Science* 367:eaaw4325. 10.1126/science.aaw4325 31896692 PMC7577560

[B46] Jovanovic T. Norrholm S. D. Davis J. Mercer K. B. Almli L. Nelson A. (2013). PAC1 receptor (ADCYAP1R1) genotype is associated with dark-enhanced startle in children. *Mol. Psychiatry* 18 742–743. 10.1038/mp.2012.98 22776899 PMC3750970

[B47] Jovanovic T. Stenson A. F. Thompson N. Clifford A. Compton A. Minton S. (2020). Impact of ADCYAP1R1 genotype on longitudinal fear conditioning in children: interaction with trauma and sex. *Neuropsychopharmacology* 45 1603–1608. 10.1038/s41386-020-0748-2 32590837 PMC7421882

[B48] Kandel E. R. Dudai Y. Mayford M. R. (2014). The molecular and systems biology of memory. *Cell* 157 163–186. 10.1016/j.cell.2014.03.001 24679534

[B49] Kessler R. C. Sonnega A. Bromet E. Hughes M. Nelson C. B. (1995). Posttraumatic stress disorder in the national comorbidity survey. *Arch. Gen. Psychiatry* 52 1048–1060. 10.1001/archpsyc.1995.03950240066012 7492257

[B50] Kilpatrick D. G. Resnick H. S. Milanak M. E. Miller M. W. Keyes K. M. Friedman M. J. (2013). National estimates of exposure to traumatic events and PTSD prevalence using DSM-IV and DSM-5 criteria. *J. Trauma Stress* 26 537–547. 10.1002/jts.21848 24151000 PMC4096796

[B51] King S. B. Lezak K. R. O’Reilly M. Toufexis D. J. Falls W. A. Braas K. (2017a). The effects of prior stress on anxiety-like responding to intra-BNST pituitary adenylate cyclase activating polypeptide in male and female rats. *Neuropsychopharmacology* 42 1679–1687. 10.1038/npp.2017.16 28106040 PMC5518896

[B52] King S. B. Toufexis D. J. Hammack S. E. (2017b). Pituitary adenylate cyclase activating polypeptide (PACAP), stress, and sex hormones. *Stress* 20 465–475. 10.1080/10253890.2017.1336535 28610473 PMC6724739

[B53] Kirry A. J. Herbst M. R. Poirier S. E. Maskeri M. M. Rothwell A. C. Twining R. C. (2018). Pituitary adenylate cyclase-activating polypeptide (PACAP) signaling in the prefrontal cortex modulates cued fear learning, but not spatial working memory, in female rats. *Neuropharmacology* 133 145–154. 10.1016/j.neuropharm.2018.01.010 29353055

[B54] Kondo T. Tominaga T. Ichikawa M. Iijima T. (1997). Differential alteration of hippocampal synaptic strength induced by pituitary adenylate cyclase activating polypeptide-38 (PACAP-38). *Neurosci. Lett.* 221 189–192. 10.1016/s0304-3940(96)13323-1 9121696

[B55] Lee J. C. Cho Y. J. Kim J. Kim N. Kang B. G. Cha C. I. (2010). Region-specific changes in the immunoreactivity of vasoactive intestinal peptide and pituitary adenylate cyclase-activating polypeptide receptors (VPAC2, and PAC1 receptor) in the aged rat brains. *Brain Res.* 1351 32–40. 10.1016/j.brainres.2010.06.048 20599818

[B56] Macdonald D. S. Weerapura M. Beazely M. A. Martin L. Czerwinski W. Roder J. C. (2005). Modulation of NMDA receptors by pituitary adenylate cyclase activating peptide in CA1 neurons requires G alpha q, protein kinase C, and activation of Src. *J. Neurosci.* 25 11374–11384. 10.1523/jneurosci.3871-05.2005 16339032 PMC6725893

[B57] Matsuyama S. Matsumoto A. Hashimoto H. Shintani N. Baba A. (2003). Impaired long-term potentiation in vivo in the dentate gyrus of pituitary adenylate cyclase-activating polypeptide (PACAP) or PACAP type 1 receptor-mutant mice. *Neuroreport* 14 2095–2098. 10.1097/00001756-200311140-00017 14600504

[B58] McGaugh J. L. (2000). Memory–a century of consolidation. *Science* 287 248–251. 10.1126/science.287.5451.248 10634773

[B59] Meloni E. G. Kaye K. T. Venkataraman A. Carlezon W. A. (2018). PACAP increases Arc/Arg 3.1 expression within the extended amygdala after fear conditioning in rats. *Neurobiol. Learn. Mem.* 157 24–34. 10.1016/j.nlm.2018.11.011 30458282 PMC6494622

[B60] Meloni E. G. Venkataraman A. Donahue R. J. Carlezon W. A. (2016). Bi-directional effects of pituitary adenylate cyclase-activating polypeptide (PACAP) on fear-related behavior and c-Fos expression after fear conditioning in rats. *Psychoneuroendocrinology* 64 12–21. 10.1016/j.psyneuen.2015.11.003 26590791 PMC4698186

[B61] Mercer K. B. Dias B. Shafer D. Maddox S. A. Mulle J. G. Hu P. (2016). Functional evaluation of a PTSD-associated genetic variant: estradiol regulation and ADCYAP1R1. *Transl. Psychiatry* 6:e978. 10.1038/tp.2016.241 27959335 PMC5290337

[B62] Merriam L. A. Baran C. N. Girard B. M. Hardwick J. C. May V. Parsons R. L. (2013). Pituitary adenylate cyclase 1 receptor internalization and endosomal signaling mediate the pituitary adenylate cyclase activating polypeptide-induced increase in guinea pig cardiac neuron excitability. *J. Neurosci.* 33 4614–4622. 10.1523/jneurosci.4999-12.2013 23467377 PMC3623015

[B63] Miles O. W. Maren S. (2019). Role of the bed nucleus of the Stria terminalis in PTSD: insights from preclinical models. *Front. Behav. Neurosci.* 13:68. 31024271 10.3389/fnbeh.2019.00068 PMC6461014

[B64] Miyata A. Arimura A. Dahl R. R. Minamino N. Uehara A. Jiang L. (1989). Isolation of a novel 38 residue-hypothalamic polypeptide which stimulates adenylate cyclase in pituitary cells. *Biochem. Biophys. Res. Commun.* 164 567–574. 10.1016/0006-291x(89)91757-9 2803320

[B65] Miyata A. Jiang L. Dahl R. D. Kitada C. Kubo K. Fujino M. (1990). Isolation of a neuropeptide corresponding to the N-terminal 27 residues of the pituitary adenylate cyclase activating polypeptide with 38 residues (PACAP38). *Biochem. Biophys. Res. Commun.* 170 643–648. 10.1016/0006-291x(90)92140-u 2383262

[B66] Moench K. M. Wellman C. L. (2017). Differential dendritic remodeling in prelimbic cortex of male and female rats during recovery from chronic stress. *Neuroscience* 357 145–159. 10.1016/j.neuroscience.2017.05.049 28596115 PMC5555043

[B67] Muller A. Joseph V. Slesinger P. A. Kleinfeld D. (2014). Cell-based reporters reveal in vivo dynamics of dopamine and norepinephrine release in murine cortex. *Nat. Methods* 11 1245–1252. 10.1038/nmeth.3151 25344639 PMC4245316

[B68] Mustafa T. (2013). Pituitary adenylate cyclase-activating polypeptide (PACAP): a master regulator in central and peripheral stress responses. *Adv. Pharmacol.* 68 445–457. 24054157 10.1016/B978-0-12-411512-5.00021-X

[B69] Nakamachi T. Ohtaki H. Seki T. Yofu S. Kagami N. Hashimoto H. (2016). PACAP suppresses dry eye signs by stimulating tear secretion. *Nat. Commun.* 7:12034. 27345595 10.1038/ncomms12034 PMC4931240

[B70] Nikolaienko O. Patil S. Eriksen M. S. Bramham C. R. (2018). Arc protein: a flexible hub for synaptic plasticity and cognition. *Semin. Cell Dev. Biol.* 77 33–42. 10.1016/j.semcdb.2017.09.006 28890419

[B71] Otto C. Kovalchuk Y. Wolfer D. P. Gass P. Martin M. Zuschratter W. (2001a). Impairment of mossy fiber long-term potentiation and associative learning in pituitary adenylate cyclase activating polypeptide type I receptor-deficient mice. *J. Neurosci.* 21 5520–5527. 10.1523/jneurosci.21-15-05520.2001 11466423 PMC6762677

[B72] Otto C. Martin M. Wolfer D. P. Lipp H. P. Maldonado R. Schutz G. (2001b). Altered emotional behavior in PACAP-type-I-receptor-deficient mice. *Brain Res. Mol. Brain Res.* 92 78–84. 10.1016/s0169-328x(01)00153-x 11483244

[B73] Pecoraro V. Sardone L. M. Chisari M. Licata F. Li Volsi G. Perciavalle V. (2017). A subnanomolar concentration of pituitary adenylate cyclase-activating polypeptide (PACAP) pre-synaptically modulates glutamatergic transmission in the rat hippocampus acting through acetylcholine. *Neuroscience* 340 551–562. 10.1016/j.neuroscience.2016.10.061 27816700

[B74] Piggins H. D. Stamp J. A. Burns J. Rusak B. Semba K. (1996). Distribution of pituitary adenylate cyclase activating polypeptide (PACAP) immunoreactivity in the hypothalamus and extended amygdala of the rat. *J. Comp. Neurol.* 376 278–294. 10.1002/(sici)1096-9861(19961209)376:2<278::aid-cne9>3.0.co;2-0 8951643

[B75] Quinn W. G. Sziber P. P. Booker R. (1979). The Drosophila memory mutant amnesiac. *Nature* 277 212–214. 10.1038/277212a0 121760

[B76] Radley J. J. Rocher A. B. Miller M. Janssen W. G. Liston C. Hof P. R. (2006). Repeated stress induces dendritic spine loss in the rat medial prefrontal cortex. *Cereb. Cortex* 16 313–320. 10.1093/cercor/bhi104 15901656

[B77] Radley J. J. Sisti H. M. Hao J. Rocher A. B. McCall T. Hof P. R. (2004). Chronic behavioral stress induces apical dendritic reorganization in pyramidal neurons of the medial prefrontal cortex. *Neuroscience* 125 1–6. 10.1016/j.neuroscience.2004.01.006 15051139

[B78] Rajbhandari A. K. Octeau C. J. Gonzalez S. Pennington Z. T. Mohamed F. Trott J. (2021). A basomedial amygdala to intercalated cells microcircuit expressing PACAP and its receptor PAC1 regulates contextual fear. *J. Neurosci.* 41 3446–3461. 10.1523/jneurosci.2564-20.2021 33637560 PMC8051692

[B79] Ramikie T. Ressler K. (2018). Mechanisms of sex differences in fear and posttraumatic stress disorder. *Biol. Psychiatry* 83 876–885. 10.1016/j.biopsych.2017.11.016 29331353

[B80] Resch J. M. Maunze B. Phillips K. A. Choi S. (2014). Inhibition of food intake by PACAP in the hypothalamic ventromedial nuclei is mediated by NMDA receptors. *Physiol. Behav.* 133 230–235. 10.1016/j.physbeh.2014.05.029 24878316 PMC4126770

[B81] Ressler K. J. Mercer K. B. Bradley B. Jovanovic T. Mahan A. Kerley K. (2011). Post-traumatic stress disorder is associated with PACAP and the PAC1 receptor. *Nature* 470 492–497. 21350482 10.1038/nature09856 PMC3046811

[B82] Ressler K. J. Paschall G. Zhou X. L. Davis M. (2002). Regulation of synaptic plasticity genes during consolidation of fear conditioning. *J. Neurosci.* 22 7892–7902. 10.1523/jneurosci.22-18-07892.2002 12223542 PMC6758105

[B83] Roberto M. Brunelli M. (2000). PACAP-38 enhances excitatory synaptic transmission in the rat hippocampal CA1 region. *Learn. Mem.* 7 303–311. 10.1101/lm.34200 11040262 PMC311348

[B84] Roberto M. Scuri R. Brunelli M. (2001). Differential effects of PACAP-38 on synaptic responses in rat hippocampal CA1 region. *Learn. Mem.* 8 265–271. 10.1101/lm.40501 11584073 PMC311380

[B85] Ross R. A. Hoeppner S. S. Hellberg S. N. O’Day E. B. Rosencrans P. L. Ressler K. J. (2020). Circulating PACAP peptide and PAC1R genotype as possible transdiagnostic biomarkers for anxiety disorders in women: a preliminary study. *Neuropsychopharmacology* 45 1125–1133. 10.1038/s41386-020-0604-4 31910434 PMC7235237

[B86] Rozeske R. R. Valerio S. Chaudun F. Herry C. (2015). Prefrontal neuronal circuits of contextual fear conditioning. *Genes Brain Behav.* 14 22–36. 10.1111/gbb.12181 25287656

[B87] Sacchetti B. Lorenzini C. A. Baldi E. Bucherelli C. Roberto M. Tassoni G. (2001). Pituitary adenylate cyclase-activating polypeptide hormone (PACAP) at very low dosages improves memory in the rat. *Neurobiol. Learn. Mem.* 76 1–6. 10.1006/nlme.2001.4014 11525248

[B88] Sauvage M. Brabet P. Holsboer F. Bockaert J. Steckler T. (2000). Mild deficits in mice lacking pituitary adenylate cyclase-activating polypeptide receptor type 1 (PAC1) performing on memory tasks. *Brain Res. Mol. Brain Res.* 84 79–89. 10.1016/s0169-328x(00)00219-9 11113534

[B89] Schmidt S. D. Myskiw J. C. Furini C. R. Schmidt B. E. Cavalcante L. E. Izquierdo I. (2015). PACAP modulates the consolidation and extinction of the contextual fear conditioning through NMDA receptors. *Neurobiol. Learn. Mem.* 118C 120–124. 10.1016/j.nlm.2014.11.014 25490058

[B90] Sehgal M. Song C. Ehlers V. L. Moyer J. R. (2013). Learning to learn - intrinsic plasticity as a metaplasticity mechanism for memory formation. *Neurobiol. Learn. Mem.* 105 186–199. 10.1016/j.nlm.2013.07.008 23871744 PMC3855019

[B91] Sehgal M. Zhou M. Lavi A. Huang S. Zhou Y. Silva A. J. (2018). Memory allocation mechanisms underlie memory linking across time. *Neurobiol. Learn. Mem.* 153 21–25. 10.1016/j.nlm.2018.02.021 29496645 PMC6064663

[B92] Shen S. Gehlert D. R. Collier D. A. (2013). PACAP and PAC1 receptor in brain development and behavior. *Neuropeptides* 47 421–430. 10.1016/j.npep.2013.10.005 24220567

[B93] Shioda S. Shuto Y. Somogyvari-Vigh A. Legradi G. Onda H. Coy D. H. (1997). Localization and gene expression of the receptor for pituitary adenylate cyclase-activating polypeptide in the rat brain. *Neurosci. Res.* 28 345–354. 9274830 10.1016/s0168-0102(97)00065-5

[B94] Spengler D. Waeber C. Pantaloni C. Holsboer F. Bockaert J. Seeburg P. H. (1993). Differential signal transduction by five splice variants of the PACAP receptor. *Nature* 365 170–175. 10.1038/365170a0 8396727

[B95] Stevens J. S. Almli L. M. Fani N. Gutman D. A. Bradley B. Norrholm S. D. (2014). PACAP receptor gene polymorphism impacts fear responses in the amygdala and hippocampus. *Proc. Natl. Acad. Sci. U. S. A.* 111 3158–3163. 10.1073/pnas.1318954111 24516127 PMC3939867

[B96] Takasaki I. Watanabe A. Yokai M. Watanabe Y. Hayakawa D. Nagashima R. (2018). In silico screening identified novel small-molecule antagonists of PAC1 receptor. *J. Pharmacol. Exp. Ther.* 365 1–18. 10.1124/jpet.117.245415 29363578

[B97] Takuma K. Maeda Y. Ago Y. Ishihama T. Takemoto K. Nakagawa A. (2014). An enriched environment ameliorates memory impairments in PACAP-deficient mice. *Behav. Brain Res.* 272 269–278. 10.1016/j.bbr.2014.07.005 25014004

[B98] Taxier L. R. Gross K. S. Frick K. M. (2020). Oestradiol as a neuromodulator of learning and memory. *Nat. Rev. Neurosci.* 21 535–550. 10.1038/s41583-020-0362-7 32879508 PMC8302223

[B99] Taylor R. D. Madsen M. G. Krause M. Sampedro-Castaneda M. Stocker M. Pedarzani P. (2014). Pituitary adenylate cyclase-activating polypeptide (PACAP) inhibits the slow afterhyperpolarizing current sIAHP in CA1 pyramidal neurons by activating multiple signaling pathways. *Hippocampus* 24 32–43. 10.1002/hipo.22201 23996525 PMC3920641

[B100] Turrel O. Goguel V. Preat T. (2018). Amnesiac is required in the adult mushroom body for memory formation. *J. Neurosci.* 38 9202–9214. 10.1523/jneurosci.0876-18.2018 30201766 PMC6705992

[B101] Twining R. C. Lepak K. Kirry A. J. Gilmartin M. R. (2020). Ventral hippocampal input to the prelimbic cortex dissociates the context from the cue association in trace fear memory. *J. Neurosci.* 40 3217–3230. 10.1523/jneurosci.1453-19.2020 32188770 PMC7159889

[B102] Uddin M. Chang S. C. Zhang C. Ressler K. Mercer K. B. Galea S. (2013). Adcyap1r1 genotype, posttraumatic stress disorder, and depression among women exposed to childhood maltreatment. *Depress. Anxiety* 30 251–258. 10.1002/da.22037 23280952 PMC4081452

[B103] Valentino R. J. Bangasser D. A. (2016). Sex-biased cellular signaling: molecular basis for sex differences in neuropsychiatric diseases. *Dialogues Clin. Neurosci.* 18 385–393. 10.31887/dcns.2016.18.4/rvalentino 28179810 PMC5286724

[B104] Varodayan F. P. Minnig M. A. Steinman M. S. Oleata C. S. Riley M. W. Sabino V. (2019). PACAP regulation of central amygdala GABAergic synapses is altered by restraint stress. *Neuropharmacology* 168:107752. 10.1016/j.neuropharm.2019.107752 31476352 PMC7048635

[B105] Vaudry D. Falluel-Morel A. Bourgault S. Basille M. Burel D. Wurtz O. (2009). Pituitary adenylate cyclase-activating polypeptide and its receptors: 20 years after the discovery. *Pharmacol. Rev.* 61 283–357. 10.1124/pr.109.001370 19805477

[B106] Wang M. Yang Y. Wang C. J. Gamo N. J. Jin L. E. Mazer J. A. (2013). NMDA receptors subserve persistent neuronal firing during working memory in dorsolateral prefrontal cortex. *Neuron* 77 736–749. 10.1016/j.neuron.2012.12.032 23439125 PMC3584418

[B107] Wellman C. L. (2001). Dendritic reorganization in pyramidal neurons in medial prefrontal cortex after chronic corticosterone administration. *J. Neurobiol.* 49 245–253. 10.1002/neu.1079 11745662

[B108] Wellman C. L. Bollinger J. L. Moench K. M. (2020). Effects of stress on the structure and function of the medial prefrontal cortex: insights from animal models. *Int. Rev. Neurobiol.* 150 129–153. 10.1016/bs.irn.2019.11.007 32204829 PMC9483990

[B109] Yaka R. He D. Y. Phamluong K. Ron D. (2003). Pituitary adenylate cyclase-activating polypeptide (PACAP(1-38)) enhances N-methyl-D-aspartate receptor function and brain-derived neurotrophic factor expression via RACK1. *J. Biol. Chem.* 278 9630–9638. 10.1074/jbc.m209141200 12524444

[B110] Yiu A. P. Mercaldo V. Yan C. Richards B. Rashid A. J. Hsiang H. L. (2014). Neurons are recruited to a memory trace based on relative neuronal excitability immediately before training. *Neuron* 83 722–735. 10.1016/j.neuron.2014.07.017 25102562

